# Supplementary figures and images for: Phylogenic classification and virulence genes profiles of uropathogenic E. coli and diarrhegenic E. coli strains isolated from community acquired infections
Source: PLoS One. 2019 Sep 12;14(9):e0222441. doi: 10.1371/journal.pone.0222441 (PMC6742363; doi:10.1371/journal.pone.0222441)

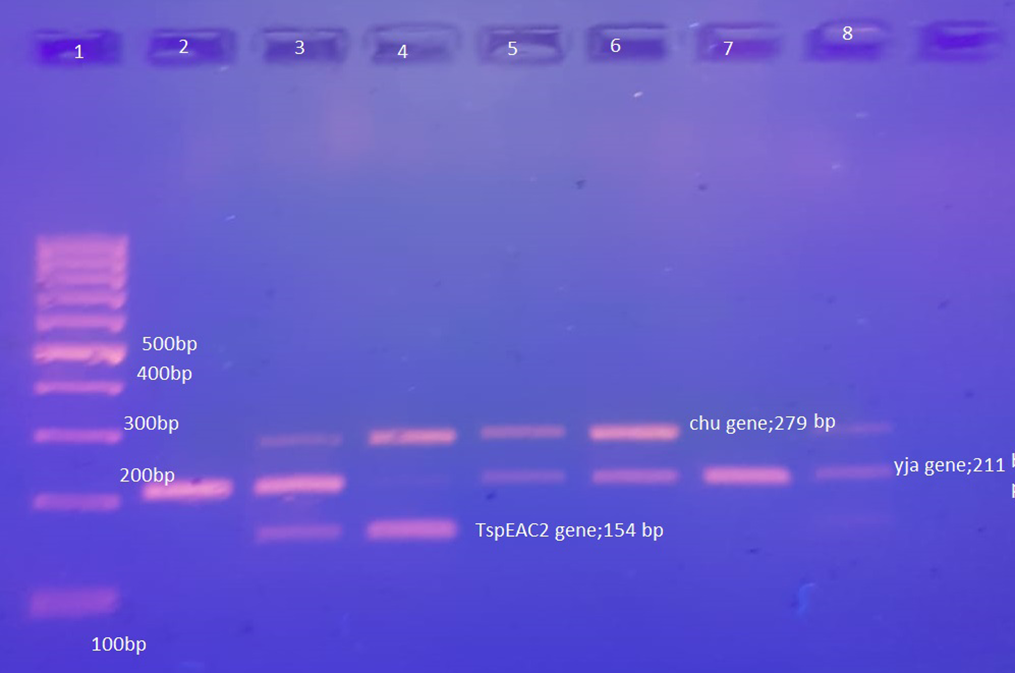

Supplement: S1 Fig — (TIF) [file pone.0222441.s001.tif]

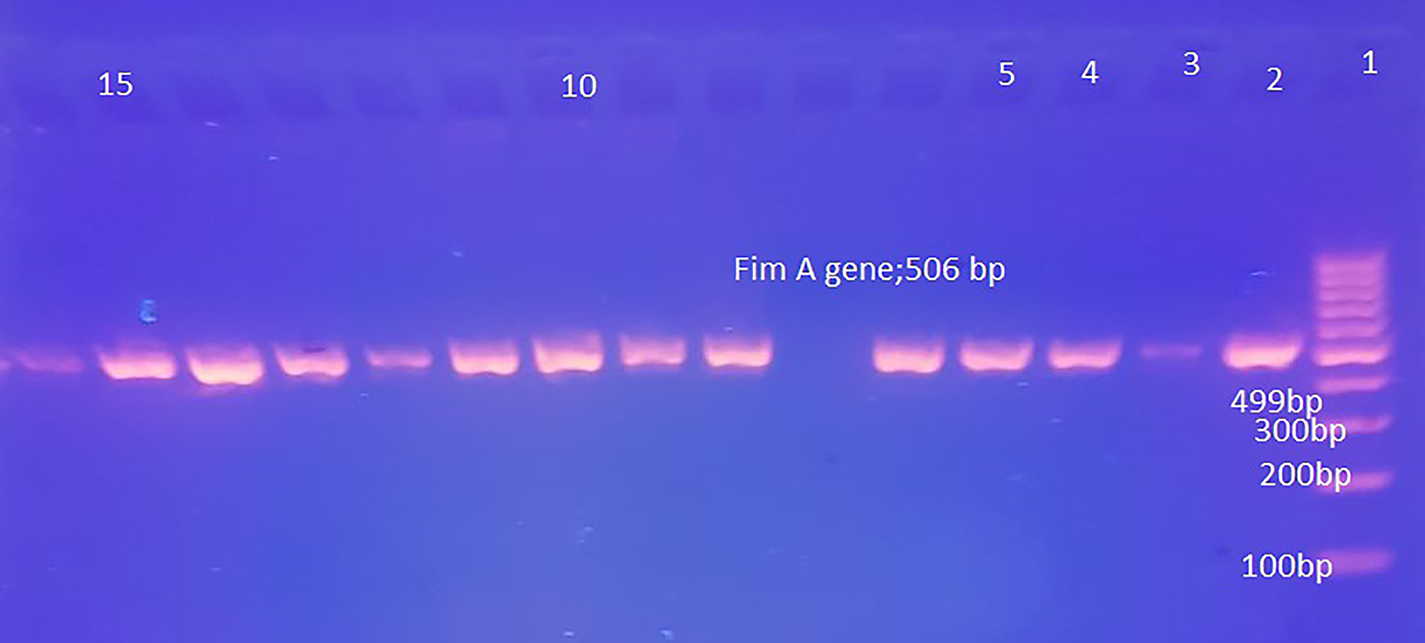

Supplement: S2 Fig — (TIF) [file pone.0222441.s002.tif]

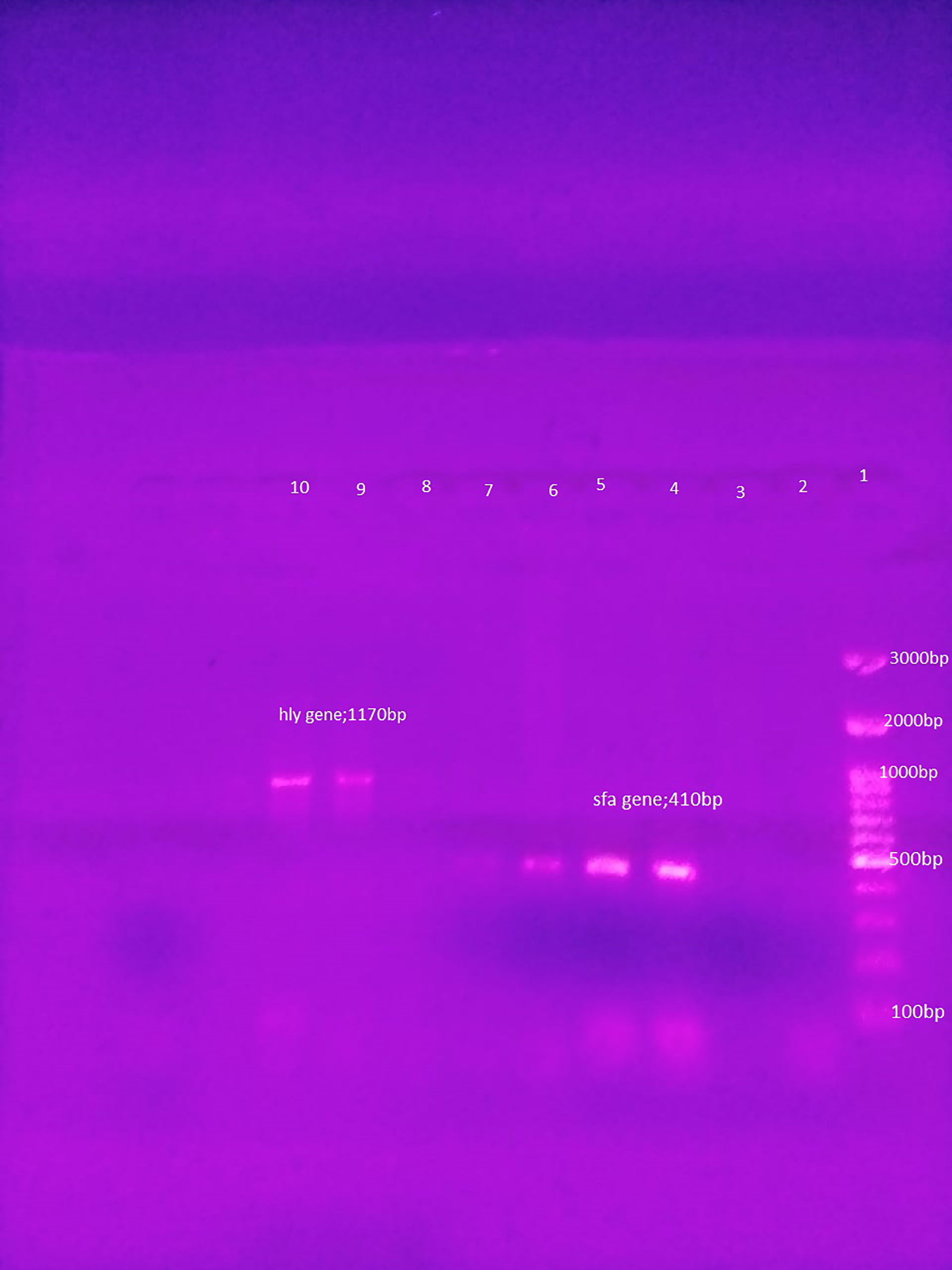

Supplement: S3 Fig — (TIF) [file pone.0222441.s003.tif]

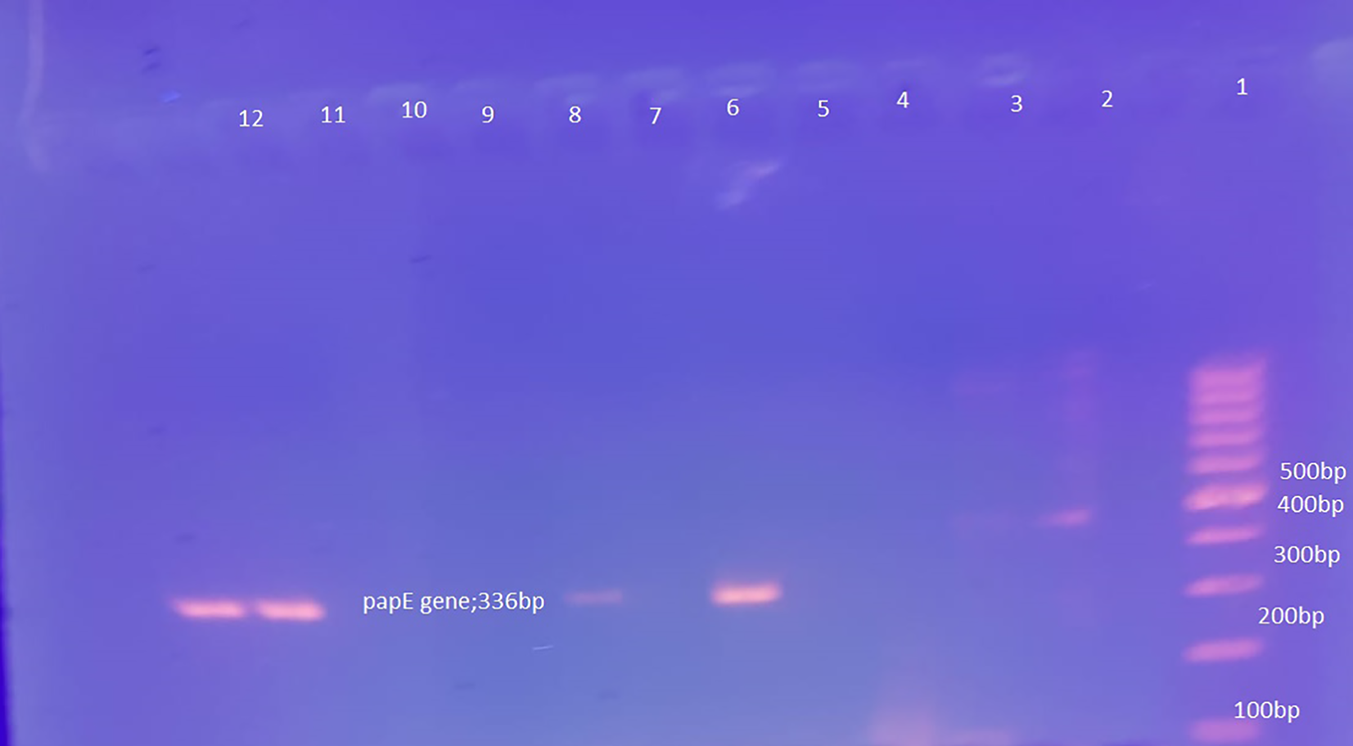

Supplement: S4 Fig — (TIF) [file pone.0222441.s004.tif]

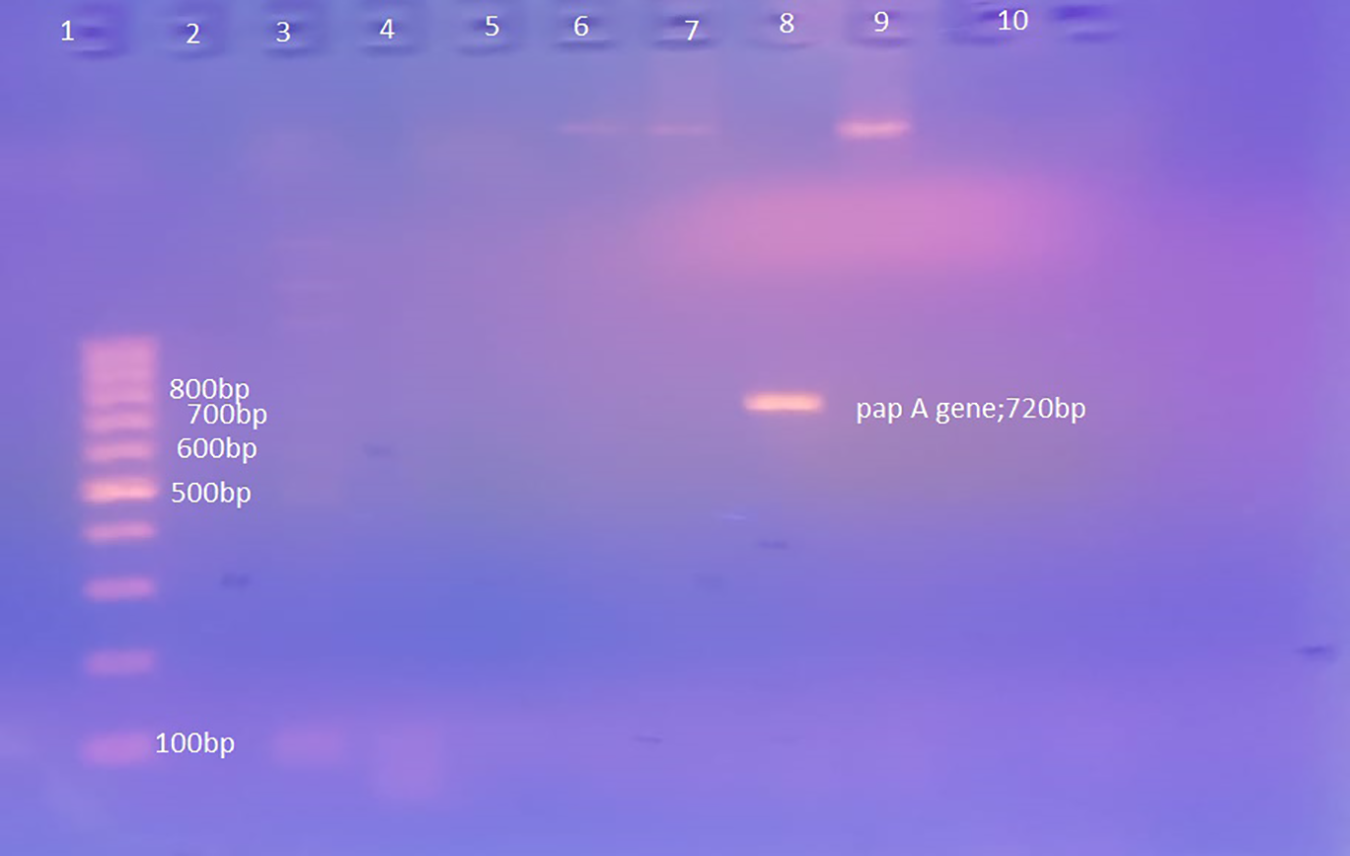

Supplement: S5 Fig — (TIF) [file pone.0222441.s005.tif]

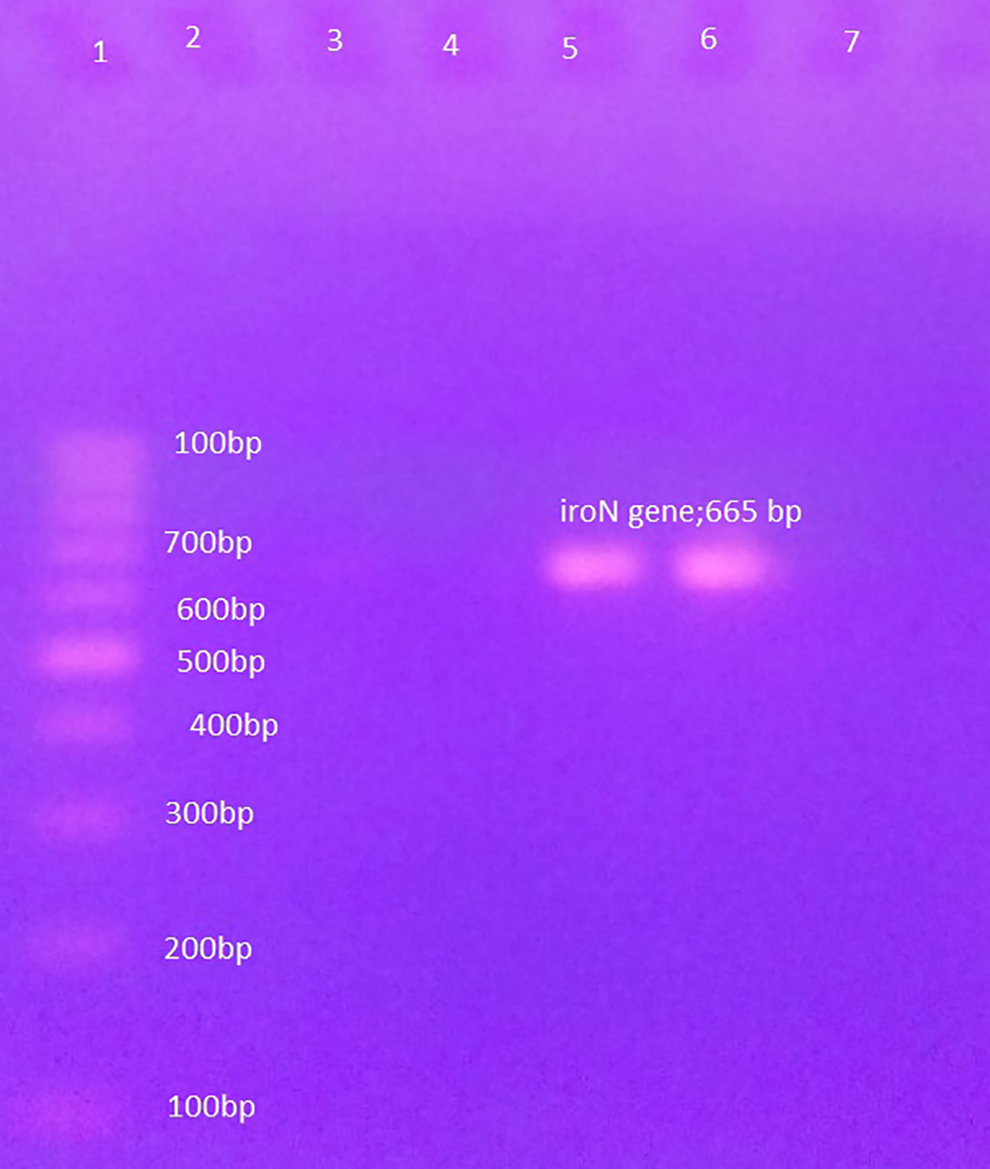

Supplement: S6 Fig — (TIF) [file pone.0222441.s006.tif]
