# Supplementary material for: Phylogenic classification and virulence genes profiles of uropathogenic E. coli and diarrhegenic E. coli strains isolated from community acquired infections
Source: PLoS One. 2019 Sep 12;14(9):e0222441. doi: 10.1371/journal.pone.0222441 (PMC6742363; doi:10.1371/journal.pone.0222441)
Supplement: Attachment — Submitted filename: Response to Reviewers.docx [file pone.0222441.s007.docx]

**Dear Prof.** Douglas MacPherson

We would like thank you, the editorial board and the two independent reviewers for their comments that improved the overall quality of our manuscript (PONE-D-19-16993
Phylogenic classification and virulence genes profiles of uropathogenic E. coli and diarrhegenic E. coli strains isolated from community acquired infections, PLOS ONE )

We are enclosing the revised manuscript with the outlined changes made in the text (highlighted). Also, our response to the comments raised by the two reviewers is outlined below in blue font after each comment.

We hope that revised article has a favorable decision towards publication in your journal.

Thank you very much.

Sincerely,

Rasha M.M. Khairy

[rashakhiry1@gmail.com](mailto:icpminia@yahoo.com); Telephone: +20-106-409-1492

**Reviewer comments 1  :**

reviewer1`
a) Virulence factors such as siderophores or adhesins, hemolysins, etc. are very common in commensal E. coli and it is not surprising that many DEC also have these genes. Having these genes doesn’t mean that the bacteria are UPEC. UPEC virulence is thought to be a multigenic phenomenon and describing all the potentially virulence genes is not very relevant.

***Response***

Bacteria are classified as intestinal pathogenic *E. coli* (IPEC), which are associated with diarrhea, and extraintestinal pathogenic *E. coli* (ExPEC), which cause infections beyond the intestinal tract) not only on the basis of their virulence properties but also the **site of infection** (the diseases that they cause),, and their host of isolation (Russo TA, Johnson JR. 2000; **Kaper, J. B.** 2005; **Kaper, J. B. et al** 2004). The study was to compare the virulence genes in both UPEC isolated from patients with UTI and DEC isolated from patients suffering from diarrhea in Egypt, where the data is **scarce.** Our isolates were recovered from diagnosed clinical infections and from pure cultures, so they associated with the diseases caused by them

The hypothesis express, that uropathogenic *E. coli* (UPEC) strains than non-pathogenic strains acquiring new virulence factors by pathogenicity islands (Mladin *et al*., 2009).

Many reports lie in the same corner with us; (Connell I, et al. 1996) who reported that Expression of type 1 fimbriae (adhesin gene *fimH* ) has been closely associated with the early development of UTI (Connell I, et al. 1996. Proc. Natl. Acad. Sci. U. S. A. 93:9827–9832.) (Johnson JR and Russo TA. 2002; Köhler CD and Dobrindt U. 2011. And others) have demonstrated that the main ExPEC virulence factors, such as P fimbriae and α-hemolysin, **are usually not present in intestinal pathogenic (IPEC) isolates.**

Lee et al. (2010) found that among commensal strains, virulence factors were **good predictors for urinary tract** or blood stream infections, while Peirano et al. (2013) defined isolates **positive for two or more of** papA and/or papC, sfa/focDE, afa/draBC, kpsM II and iutA as **ExPEC.**

**Our results agree with that UPEC virulence is thought to be a multigenic phenomenon as we found UPEC isolates exhibited 20 Ec** patterns while DEC isolates exhibited 5 Ec patterns only. Combined virulence patterns are in UPEC. Some strains of *E. coli* can diverge from their commensal cohorts, taking on a more pathogenic nature. These strains acquire specific virulence factors (horizontal transfer of transposons, plasmids, bacteriophages, and pathogenicity islands).

- We also found that some virulence genes are not common in DEC isolates. *sfa* and *papE* genes were detected in UPEC isolates in (44/103, 42.7%) and (17/103, 17.1%) respectively but were not detected in DEC isolates at all.

VGs are ideal targets for determining the pathogenic potential of a given E. coli isolate (Kuhnert P, Boerlin P, Frey J. 2000. FEMS Microbiol. Rev. 24:107–117)

b) It is not clear how they obtained the EAEC isolates; if they obtained from fecal cultures is very odd that all diarrheic samples were positive to EAEC. Normally you obtain 5-20% of different DEC pathotypes. If strains were obtained from a collection, why the authors concentrate in EAEC and ignore DEC pathotypes?

We concentrated in EAEC because it is the most common type in Egypt (Ali et al., 2014; El Gamel et al., 2015). Other types are less frequent and their number in most of collections is not representative.

c) Study group is not described either for UTI patients or for diarrhea patients.

We thank the reviewer for comment. The revised manuscript included the reviewer's suggestion.

d) Many reports have shown that some intestinal pathotypes (DEC) can also cause urinary infection DAEC and EAEC, it is not a novel finding.

There are no studies done in Egypt to associate between DEC isolates and UPEC isolates. So we tried to study this issue in a new geographical area. Only one or 2 reports have studied the virulence factors of DEC (*eae, bfp*) in UTI.

The pathogenic E. coli strains use a complex multistep mechanism of pathogenesis involving a number of virulence factors depending upon the pathotype, which consists of attachment, host cell surface modification, invasin, a variety of toxins, and secretion systems which eventually lead toxins to the target host cells (kapper. 2004). Thus, VGs are ideal targets for determining the pathogenic potential of a given E. coli isolate (Kuhnert P, Boerlin P, Frey J. 2000).

e) The association of some DEC or UPEC strains in this study with Clermon’s phylogenetic groups is not uncommon. Virulence genes are transferred horizontally.

The phylogenetic grouping was done to identify the prevalence of different phylogenetic groups in Egypt and comparing between UPEC and DEC isolates to increase the knowledge about the geographical distributions of these types. In contrast to most reports, phylogenetic group A was the most prevalent in both UPEC and DEC strains, followed by B2 group. The predominance of phylogenetic group A which is usually associated with commensal strains suggesting that the gastrointestinal tract is the main source of strains that may be colonize the urinary tract. This increases and highlights the emergence of virulent phylogroups A and B1. This result was similar to some previous studies (Rahman et al., 2017). Although other studies showed the more prevalence of phylogenetic groups B2 and D (ElSayed Gawad et al., 2018). It was surprising that B1 type was not detected in DEC isolates at all.

***Minor problems***
a) The manuscript contains many grammatical errors.

We thank the reviewer for comment. The revised manuscript included the reviewer's suggestion
b) The discussion section is too long

We thank the reviewer for comment. The revised manuscript included the reviewer's suggestion
c) Figures showing distributions of strains in Clermon’s phylogenetic groups are unnecessary.

We thank the reviewer for comment. The revised manuscript included the reviewer's suggestion
d) We thank the reviewer for comment. The revised manuscript included the reviewer's suggestion

Lines 39-41 text improvement is needed

Done

e) 107-126 Confidence intervals 95% are not reported for frequency data. There is not p value reported next to percentage of positiveness.

We thank the reviewer for comment. The revised manuscript included the reviewer's suggestion

f) Table 2 and table 3 could be join in one.

We thank the reviewer for comment. The revised manuscript included the reviewer's suggestion

g) 149-151 Use UPEC instead of ExPEC because you are working only with urine infections associated isolates.

Ok

h) 159 Country names must be written with uppercase

Ok
i) 157-161 Improve English writing

We thank the reviewer for comment. The revised manuscript included the reviewer's suggestion
j) The format of bibliographical references should be checked
We thank the reviewer for comment. The revised manuscript included the reviewer's suggestion

**Reviewer #2: Interesting article. It brings information that can be taken into account in future studies and analysis at a global level. Evaluations within the framework of general data are, however, rather geared towards parts of the globe and less in Europe (eg could be seen** [**doi.org/10.1155/2019/5712371**](http://doi.org/10.1155/2019/5712371)**). Please review the English language. I've seen some errors to mention just one (page 4, 77, enteroaggragative). Please review the bibliography - it is not unitary written.**

 We thank the reviewer for comment. The revised manuscript included the reviewer's suggestion

6
